# The Olfactory Organ Is a Unique Site for Neutrophils in the Brain

**DOI:** 10.3389/fimmu.2022.881702

**Published:** 2022-05-27

**Authors:** M. Fernanda Palominos, Cristian Calfún, Gino Nardocci, Danissa Candia, Jorge Torres-Paz, Kathleen E. Whitlock

**Affiliations:** ^1^ Centro Interdisciplinario de Neurociencia de Valparaíso (CINV), Universidad de Valparaíso, Valparaíso, Chile; ^2^ Instituto de Neurociencia, Universidad de Valparaíso, Valparaíso, Chile; ^3^ Faculty of Medicine, Center for Biomedical Research and Innovation (CIIB), Universidad de los Andes, Santiago, Chile; ^4^ IMPACT, Center of Interventional Medicine for Precision and Advanced Cellular Therapy, Santiago, Chile

**Keywords:** olfactory sensory neurons (OSNs), olfactory Bulb (OB), olfactory imprinting, neutrophil brain migration, *mpx:Dendra2*, bromodeoxyuridine (BrdU)

## Abstract

In the vertebrate olfactory tract new neurons are continuously produced throughout life. It is widely believed that neurogenesis contributes to learning and memory and can be regulated by immune signaling molecules. Proteins originally identified in the immune system have subsequently been localized to the developing and adult nervous system. Previously, we have shown that olfactory imprinting, a specific type of long-term memory, is correlated with a transcriptional response in the olfactory organs that include up-regulation of genes associated with the immune system. To better understand the immune architecture of the olfactory organs we made use of cell-specific fluorescent reporter lines in dissected, intact adult brains of zebrafish to examine the association of the olfactory sensory neurons with neutrophils and blood-lymphatic vasculature. Surprisingly, the olfactory organs contained the only neutrophil populations observed in the brain; these neutrophils were localized in the neural epithelia and were associated with the extensive blood vasculature of the olfactory organs. Damage to the olfactory epithelia resulted in a rapid increase of neutrophils both within the olfactory organs as well as the central nervous system. Analysis of cell division during and after damage showed an increase in BrdU labeling in the neural epithelia and a subset of the neutrophils. Our results reveal a unique population of neutrophils in the olfactory organs that are associated with both the olfactory epithelia and the lymphatic vasculature suggesting a dual olfactory-immune function for this unique sensory system.

## Introduction

### Neurons and Neutrophils

In vertebrates the olfactory sensory neurons (OSNs), a group of continually renewing neurons located in the olfactory epithelium (OE), extend their axons across the cribriform plate where they make their first synapses in the olfactory bulb (OB) (1, 2). Odor mediated social interactions in fishes (3, 4) and rodents (5) use major histocompatibility complex (MHC) peptides that may also interact with olfactory receptors and T cell antigen receptors to link social cues with assessments of the health state of the animal (6). Additionally, “olfactory imprinting” characterized in salmon by odor-driven migration to their natal stream (7) has more recently been shown to be based on the ability to discern relatedness among conspecifics through detection of MHC peptides as evidenced by the long term memory of genetically determined kin signals (8) and the navigation of coral reef fish to their home reef (9).

We have previously used zebrafish as a model system to study the cellular basis of olfactory memory in the peripheral nervous system, namely the olfactory epithelia, and showed that zebrafish retain into adulthood memories of odors experienced as juveniles. To identify genes involved in the formation of olfactory memory we then analyzed the genomic response of the adult OE in olfactory imprinted fish by both microarray and RNAseq analyses (10–12). In addition to known genes expressed in the OE, we found genes specific to both the innate and the adaptive immune systems (13) prompting us to investigate the “immune architecture” of the OE that may contribute to long-term memory.

Neutrophils, the most abundant type of white blood cells in mammals, are now known to play a key role in both the innate and the adaptive immune response (14–16), where they can rapidly migrate to lymph nodes *via* not only on the blood vasculature and interstitial tissues, but also *via* afferent lymphatics of inflamed tissues (17–20). Under normal conditions, neutrophils are scarce in the central nervous system (CNS), where the brain–blood barrier (BBB) prevents their migration into the brain parenchyma and cerebrospinal fluid. Conditions of neuroinflammation and injury-induced damage to the BBB are associated with the infiltration to the CNS of neutrophils (21–23). Most recently, in mouse, neutrophils have been shown to infiltrate into the olfactory organs and express proinflammatory genes in response to inflammation, but then slowly initiate expression of neurogenesis-related genes (24, 25). Using zebrafish as a model system we have recently shown that neutrophils populate the developing olfactory organs and use the blood vasculature to migrate to the olfactory organ in response to injury (26), yet little is known about the interactions of neutrophils in the adult olfactory organ.

### The Adult Olfactory Organ Blood-Lymphatic System

The connection between the OE and the OB is part of a complex neural and immune interface that includes flow of cerebral spinal fluid (CSF) and interstitial fluid (ISF) from the subarachnoid space toward the nasal mucosa. Evidence supporting the existence of a connection between the subarachnoid space of the brain and cervical lymph nodes *via* the nasal mucosa was first proposed over a century ago (for review see: (27, 28). Subsequent studies in mammals using labeled tracers confirmed a drainage route from the cranial subarachnoid space through the olfactory pathway and leaving the nasal mucosa *via* terminal lymphatics or blood capillaries (29, 30). Thus immunogenic material and immune cells from the CNS could pass to immune organs outside the brain *via* the olfactory epithelia. Of particular interest are the nasopharynx-associated lymphoid tissues (NALT), a term used in mammals to describe the network of lymphoid tissue in the pharynx and palate (tonsils). Teleost fish lack organized lymphoid structures such as tonsils yet a recent study suggested the presence of a NALT-like diffuse network of lymphoid and myeloid cells scattered both intraepithelial and in the *lamina propria* of the fish olfactory organ (31).

More recently, the “re-discovery” of lymphatic vasculature associated with the meninges of the CNS of mammals (32–35) and of zebrafish (36, 37) has led to a renewed interest in immune trafficking in the nervous system *via* the sinus-associated meningeal lymphatic vessels and/or *via* cribriform plate and nasal lymphatics into cervical lymph nodes (38–40). To date, and in spite of over a century of reports on “brain drainage” through the olfactory system/nasal mucosa and the expanded knowledge of lymphatic vasculature in the vertebrate brain, there are no detailed descriptions of the lymphatic vasculature (LV) in the olfactory organ.

Here, we show for the first time in zebrafish that the olfactory organs of the adult animal contain neutrophils that are activated upon injury with the suggestion that injury, and perhaps other neuroinflammatory stimuli, allow for neutrophil trafficking across the BBB *via* the blood-lymphatic vasculature associated with the olfactory nerve. Better understanding of the olfactory neural-immune architecture will allow us to dissect the cellular basis of olfactory memory and immunity.

## Material and Methods

### Animals

Zebrafish were maintained in a re-circulating system (Aquatic Habitats Inc, Apopka, FL) at 28°C on a light-dark cycle of 14 and 10 hours respectively. All fish were maintained in the Whitlock Fish Facility at the Universidad de Valparaiso. Wild-type (WT) fish of the Cornell strain (derived from Oregon AB) were used. All protocols and procedures employed were reviewed and approved by the Institutional Committee of Bioethics for Research with Experimental Animals, University of Valparaiso (#BA084-2016). Adults used in the study were 12-16 months of age. The following transgenic lines were used to visualize specific cell types: *Tg(BACmpx:gfp)^i114^, Tg(mpx:GFP) (* 41 *)*; (*Tg(fli1a:EGFP)^y1^ Tg(fli1a:EGFP*; (42); *Tg(−5.2lyve1b:DsRed)^nz101^, Tg(2lyve1b:DsRed) Tg(−5.2lyve1b:EGFP)^nz151^ Tg*(*lyve1b:EGFP*), (43); *Tg(gata1a:DsRed)^sd2^ Tg*(*gata1a:DsRed*) (44), *Tg(pomp^2k^:gap-YFP)^rw032a^*, *Tg(omp:YFP); Tg(pomp^2k^:lyn-mRFP)^rw035a^ Tg(omp:RFP*) (45); *Tg(six4b:mCh)*, (46); *Tg(mpx:Dendra2)* (47).

### Copper Exposure

Initial dose response analysis was performed based on previous work in zebrafish and salmon (48, 49). A stock solution of 10 mM CuSO_4_ was diluted in system water for a final concentration of 10 uM CuSO_4_.

### Immunocytochemistry and Cell Labeling

Dissected adult brains were fixed in 4% PFA in 0.1M phosphate buffer 0.4M pH 7.3), or 1X phosphate-buffered saline PBS pH 7.4. Brains were rinsed three times in phosphate buffer or PBS, permeabilized in acetone at -20°C for 10 minutes and then incubated for two hours in blocking solution (10 mg/ml BSA, 1% DMSO, 0.5% Triton X-100 (Sigma) and 4% normal goat serum in 0.1M phosphate buffer or 1X PBS). Primary antibodies used were anti-RFP (rabbit 1:250, Life Technologies), anti-GFP (mouse 1:500, Life Technologies), anti-GFP (rabbit 1:500, Invitrogen), anti-DsRed (mouse 1:500, Santa Cruz Biotechnology), anti-HuC/D (rabbit 1:500, Invitrogen) and anti-BrdU (rabbit 1:250,Invitrogen). Adult brains were incubated with the primary antibody for up to a week. After washes, tissues were incubated overnight in experiment dependent secondary antibodies: Dylight 488 conjugated anti-mouse antibody (goat 1:500, Jackson Immuno Research), Alexa Fluor 488 conjugated anti-rabbit antibody (goat 1:1000, Molecular Probes), Alexa Fluor 568 conjugated anti-rabbit antibody (goat 1:1000, Molecular Probes), Alexa Fluor 568 conjugated anti-mouse antibody (goat 1:1000, Molecular Probes), Dylight 650 conjugated anti-rabbit antibody (goat 1:500, Jackson Immuno Research), Alexa Fluor 350 conjugated anti-rabbit antibody (goat 1:1000, Molecular Probes). Tissues were then rinsed in 0.1M phosphate buffer or 1X PBS with 1% DMSO, stained for DAPI (1 μg/ml, Sigma), washed in 0.1M phosphate buffer or 1X PBS and mounted in 1.5% low melting temperature agarose (Sigma) in an Attofluor Chamber for subsequent imaging (see below).

### BrdU Labeling

For each experiment nine adult fish were first housed overnight in 1.5 liter tanks containing 10 mM BrdU in system water. The next morning three fish were transferred to a new 1.5-liter tank with system water (control) and six fish were transferred to a new 1.5 liter tank with system water containing 10 μM CuSO_4_, and allowed to swim freely (4 hours). All control fish (3) and half of copper-exposed fish (3) were then anesthetized, sacrificed and heads fixed overnight in 4% PFA/1X PBS. The other half of copper-exposed fish (3) were transferred to a clean 1.5-liter tank, filled with system water, and allowed to recover. The next day, these fish were anesthetized, sacrificed and fixed as described above. After fixation, heads were incubated in EDTA (0.2 M, pH 7.5) for three days at 4°C and brains dissected in sterile 1X PBS and pre treated in 2 M HCl for 30 minutes at 37°C. Immunocytochemistry was performed as described in Immunocytochemistry & Cell Labeling. For imaging, whole adult brains were mounted on 2% low melting temperature Agarose, and OE were mounted between coverslips, as described above. The removal of brains from the skull with the OO still attached is a difficult dissection because the OSN axons pass through the cribriform plate to arrive in the OB. Therefore it was not always possible to have a preparation with both OE still connected to the brain.

### Cryosectioning

Fish were euthanized and heads were fixed overnight in 4% PFA at 4°C and decalcified in EDTA (0.2 M, pH 7.6) for 3 days, and later embedded in 1.5% agarose/5% sucrose blocks and submerged in 30% sucrose for 3 days at 4°C. Blocks were frozen (-20°C) with O.C.T. Compound (Tissue Tek^®^) and sectioned (25 μm) using a cryostat.

For flat mounting of the olfactory epithelia, olfactory rosettes were dissected after immunohistochemistry or staining, and mounted with the caudal side down on Poly-L-Lysine coated slides between triple 22x22 coverslip bridges and covered in VECTASHIELD^®^ Antifade Mounting Media (Vector laboratories).

### Imaging and Image Analysis

*Microscopy:* Fluorescent images were taken using a Spinning Disc microscope Olympus BX-DSU (Olympus Corporation, Shinjuku-ku, Tokyo, Japan) and acquired with ORCA IR2 Hamamatsu camera (Hamamatsu Photonics, Higashi-ku, Hamamatsu City, Japan). Images were acquired using the Olympus CellR software (Olympus Soft Imaging Solutions, Munich, Germany). Some images were also obtained using a confocal laser scanning microscope (Nikon C1 Plus; Nikon, Tokyo, Japan). Images were then deconvoluted in AutoQuantX 2.2.2 (Media Cybernetics, Bethesda, MD, USA) and processed using FIJI (National Institute of Health, Bethesda, Maryland, USA; (50) and CellProfiler (51).

### Image Analyses

*Neutrophils*: Only neutrophils within the boundaries of the olfactory organs in adults were counted and the values were given as the average of total number of mpx:GFP positive with standard deviation. Values given for paired sensory structure are a sum of the individual sensory tissues.

To analyze the distribution of mpx:GFP^+^ neutrophils from both whole adult brains and flat-mounted olfactory rosettes, images were filtered by size (6-30 μm) and pixel intensity, and then counted using CellProfiler available Pipelines (51). For quantification of neutrophils in different regions of the OE, sensory (ss) versus non-sensory (ns) regions were separated using *Tg(omp:RFP)* animals or anti-HuC/D labeling as neuronal markers. We grouped the ns region with the epineurial extensions (EN) wrapping the OE. The percent of total neutrophils is the number of GFP cells in ss or ns regions, divided by total (sum of all GFP positive cells in ss, ns and EN). BrdU nuclei were detected by filtering size between 2-5 μm and co-localization between BrdU and neutrophils was done using “Co-localization” Pipeline in CellProfiler (51).

The circularity index of each neutrophil was calculated using Analyze Particles in FIJI (National Institute of Health, Bethesda, Maryland, USA; (50). Neutrophils were size-filtered and values were graphed according frequency of distribution.

*BV/LV vessel density*. Density is defined by the ratio of the area positive for fli1a:EGFP (BV) and lyve1b:DsRed (LV) over the total dorsal telencephalic or the olfactory system area (which includes both the OE and OB). Protocol adapted from (52).

### Photoconversion

The protocol “Labeling cells with photoconvertible fluorescent proteins in zebrafish” was adapted for adult fish. Homozygote (2.5 months) *Tg(mpx:Dendra2)* (ZDB-TGCONSTRCT-110209-4) fish expressing Dendra2 (original 490/507; photoconverted 553/573; octocoral *Dendronephthya* sp. (47, 53) were used. Fish were anesthetized using Tricaine (40mg/L) in tank water and positioned using small wet sponges. Using the 10x objective, olfactory organs were illuminated by the border of the visual field when the size of the pinhole on the Leica DMR microscope (Leica Microsystems CMS GmbH, Wetzlar, Germany) was adjusted. Fish were checked for Mpx : Dendra2 positive cells (green) in the olfactory organ before initiated the photoconversion. The photoconversion, as judged by the appearance of conversion to red fluorescence, required ~90 seconds. Fish were allowed to recover and then exposed to copper (see above). Images were acquired using a Leica DFC 480 camera (Leica Microsystems Ltd, Heerbrugg, Switzerland), and processed with the Leica Application Suite 2.3.3 software (Leica Microsystems Ltd).

**Statistics.** Data are presented as means ± standard deviations. Experiments number and statistical analysis were done using Prism 9 (Graphpad), and are indicated in each figure legend. Unpaired Student’s t-tests were performed unless otherwise indicated. P values are indicated as follows: *P, 0.05, **P, 0.01, ***P, 0.001.

### GO Analysis of Data From Imprinted Adult OO

Using data collected from adult olfactory organs of treated (PEA Imprinted) and control animals (11) a GO analysis was performed. The quality of the sequencing reaction was analyzed with the FastQC software (v0.11.9) (https://www.bioinformatics.babraham.ac.uk/projects/fastqc/) and trimmed with Trim_galore (v0.6.4) (https://www.bioinformatics.babraham.ac.uk/projects/trim_galore/). Reads were aligned to the Zv9 reference genome using Bowtie2 software with standard settings (doi:10.1038/nmeth.1923). After alignment, the gene abundance was determined using the HTseq software (v0.11.2) (https://htseq.readthedocs.io/en/master/) (doi:10.1093/bioinformatics/btu638) to calculate the raw reads number for each gene. Differential gene expression was estimated using DESeq2 (v1.24) (10.1186/s13059-014-0550-8) within SARTools R package (v1.6.9) (http://dx.doi.org/10.1371/journal.pone.0157022). Gene Ontology (GO) analysis were carried out using the GO stat R package (10.1093/bioinformatics/bth088). The data have been deposited in NCBI’s Gene Expression Omnibus (54) and are accessible through GEO Series accession number GSE196102 (https://www.ncbi.nlm.nih.gov/geo/query/acc.cgi?acc=GSE196102).

## Results

### Neutrophil Populations in the Adult Olfactory Organ

**Figure 1:**
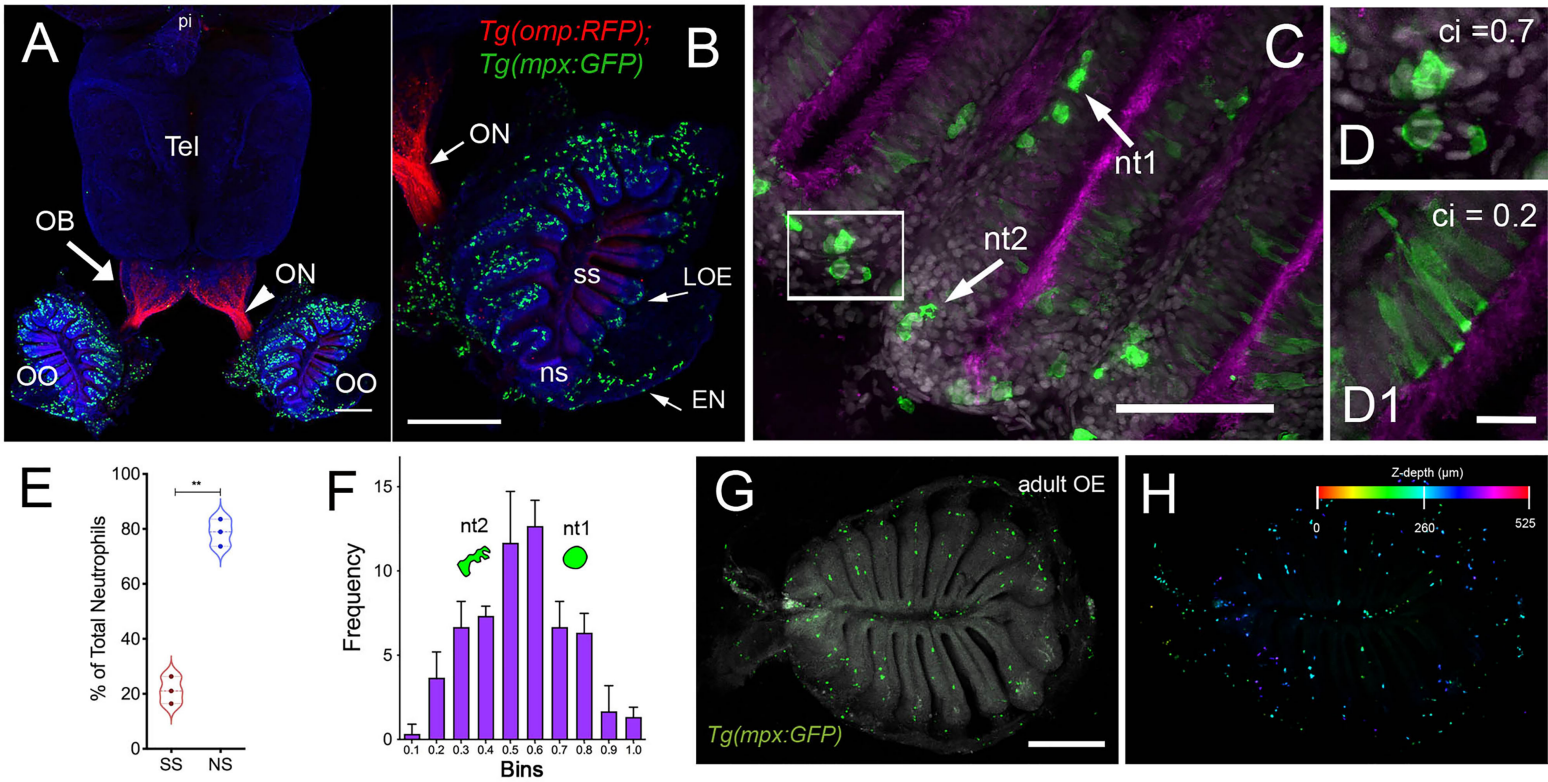
Neutrophils are found only in the olfactory organs of the adult brain. **(A)** Wholemount brain of *Tg(omp:RFP);Tg(mpx:GFP)* adult: neutrophils (green) are only present in the OO (OE/EN); Tel: telencephalon; pi: pineal. Scale bars A, B = 200 μm. **(B)** OO (from A) contains a large population of neutrophils (green, *n*=487 neutrophils). omp:RFP^+^ OSNs are located only in sensory epithelia (ss, red) not in non-sensory epithelia (ns). ON: olfactory nerve. **(C)** Neutrophils with a rounded shape, (nt1, arrow: circularity index 0.7 or greater) and amoeboid shape (nt2, arrow: circularity index of 0.4-0.6; **F**) were observed in the LOE. **(D)** Neutrophils with an amoeboid shape (nt2, arrow) were located throughout the OE and EN. **(D1)** Sustentacular-like cells **(D1)**, circularity index 0.2, lie at ns-ss epithelia interface (see **Figure 2**). **(E)** Total number of neutrophils in the OO. The non-sensory (ns) tissues (respiratory epithelia + NE, blue) have more neutrophils than sensory epithelia (ss, red), n= 3 OE from 3 different fish. **(E)** **indicates significant statistical differences between means with a p-value < 0.002, paired t-test.. **(F)** Frequency distribution of nt1 and nt2 cells (*n*= 53 neutrophils from brain shown in **C**). **(G)** Maximal projection of whole mount *Tg(mpx:GFP)* adult OE: Neutrophils (green); autofluorescence (gray). **(H)** Neutrophils (from *G*) were color-coded based on **(H)** Z-stack depth. Total depth= 550μm. Scale bars **A, B** = 200 μm; **C** = 60 μm; **D**, **D1** = 20 μm; **G, H** =100 μm. **(A, B)** 9 brains imaged; **(C, D)** 6 brains sectioned.

**Figure 2:**
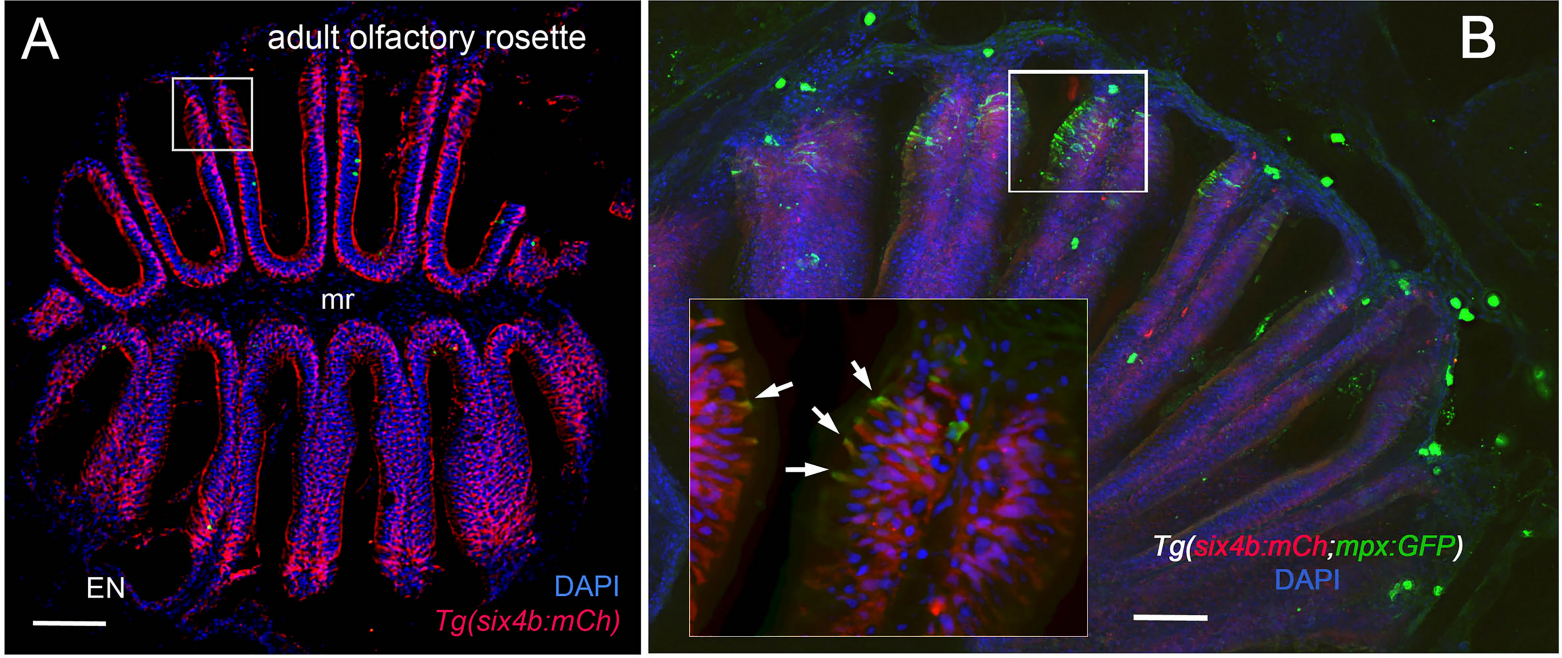
Sustentacular cells in the OE are associated with markers for neutrophils. **(A, B)** Cryosections of adult olfactory epithelia. **(A)** Adult olfactory rosette (OE) from Tg(six4b:mCh) line showing sustentacular cells (red) that are distributed within the lamellae of the OE where some areas have denser clusters (boxed area). EN: Epineurium; mr: midline raphe. Scale bar = 100 μm. **(B)** Sectioned OE of Tg(six4b:mCh;mpx:GFP) Six4b:mCh+ SCs (red) and mpx:GFP+ neutrophils(green) are found in the respiratory OE near the tips of the lamellae (boxed area). Scale bar = 50 μm. Inset B:. A small subset on cells are positive are both mpx:GFP+ and Six4b:mCh+ positive (arrows). **(A)** 1 sectioned brain, **(B)** 2 sectioned brains.

Using RNA sequencing data from our original studies on the genomic responses of the peripheral olfactory organ in imprinted versus control animals (11, 13), a GO analysis was performed (GEO Series accession number GSE196102) which revealed significant correlation between the formation of olfactory memory and up-regulation of immune specific genes including but not limited to *immunoglobulin light 3 variable 5* (*igl3v5*), *immunoglobulin heavy variable 2-1* (*ighv2-1*), *immunoglobulin heavy variable 5-3* (*ighv5-3*), and *immunoglobulin heavy constant delta* (*ighd*), of the adaptive immune system, and *toll-like receptor 19* (*tlr19*) of the innate immune system. Recently neutrophils have been shown to interact with cells of the adaptive immune system [see for review (55)] including production of Ig heavy and light chains in a subset of monocytes and macrophage (56), where the expression of IgM heavy chains and IgK light chains in human monocytes and neutrophils support Ig production by myeloid cells (57, 58). Because the adaptive immune system is responsible for immunological memory and reporter lines for neutrophils were readily available, we initiated a characterization of the neutrophils and their potential association with the olfactory organ in adult animals. We used *Tg(omp:RFP);Tg(mpx:GFP*) animals to visualize olfactory sensory neurons (OSNs, red) and neutrophils (green), in fixed whole mount brains. The omp:RFP^+^ OSNs (**Figures 1A, B**, red, ss, red) are in the central sensory epithelia (ss) and peripheral regions of the lamellae are made up of the non-sensory epithelia (ns) (**Figure 1B**, ns). Surprisingly, we observed neutrophils only in the OO of adult brains (**Figures 1A, B** green). Neutrophils were localized in the fingerlike lamellae (LOE) of the OE, predominantly associated with the epineurium (EN) wrapping around the OE (**Figure 1B**) where the tips of the LOE are connected to the EN (**Figure 1B**, EN, LOE, blue; **Figure 2**). Analysis of the distribution of GFP-positive neutrophils revealed that they were located primarily in the ns epithelia and EN with many fewer neutrophils in the ss epithelia (**Figures 1B, E**). Within the OE/EN there were three morphologically distinct mpx:GFP^+^ cells (**Figures 1C, D, F**): Neutrophils with rounded shape (**Figure 1C**, green, nt1) were associated with the basal OE, while neutrophils with amoeboid like morphology (**Figure 1C**, green, nt2, D, ci=0.7) were present in the tips of the LOE and EN, although this distribution changed in response to damage of the OE (see below). In sectioned OE tissue the columnar shaped mpx:GFP^+^ cells (**Figure 1D1**, green) were morphologically similar to sustentacular cells of the OE visualized with the *Tg(six4b:mCh)* reporter line (59); **Figures 2A, B** red). We next generated *Tg(six4b:mCh;mpx:GFP*) animals and let them grow to adulthood. Sectioned tissue of the OE revealed a subset of six4b:mCh+ cells that were also mpx:GFP+ (**Figure 2B**, inset). These cells lie at the interface of ns epithelia (**Figure 2B**, boxed area) in the distal LOE and further studies are needed to carefully characterize this class of mpx:GFP^+^ cells.

To confirm that the neutrophils observed in the whole mount OE (**Figure 1G**, green) were within the OE as opposed to coating superficial layers, a z-stack analysis was performed (**Figures 1G, H**) showing that the mpx:GFP^+^ cells are within the OE tissue. Thus the adult OOs are unique because they are the only regions of the adult brain where neutrophils are found under normal conditions.

### Neutrophil Response to Damage in the Adult Olfactory Sensory System

**Figure 3:**
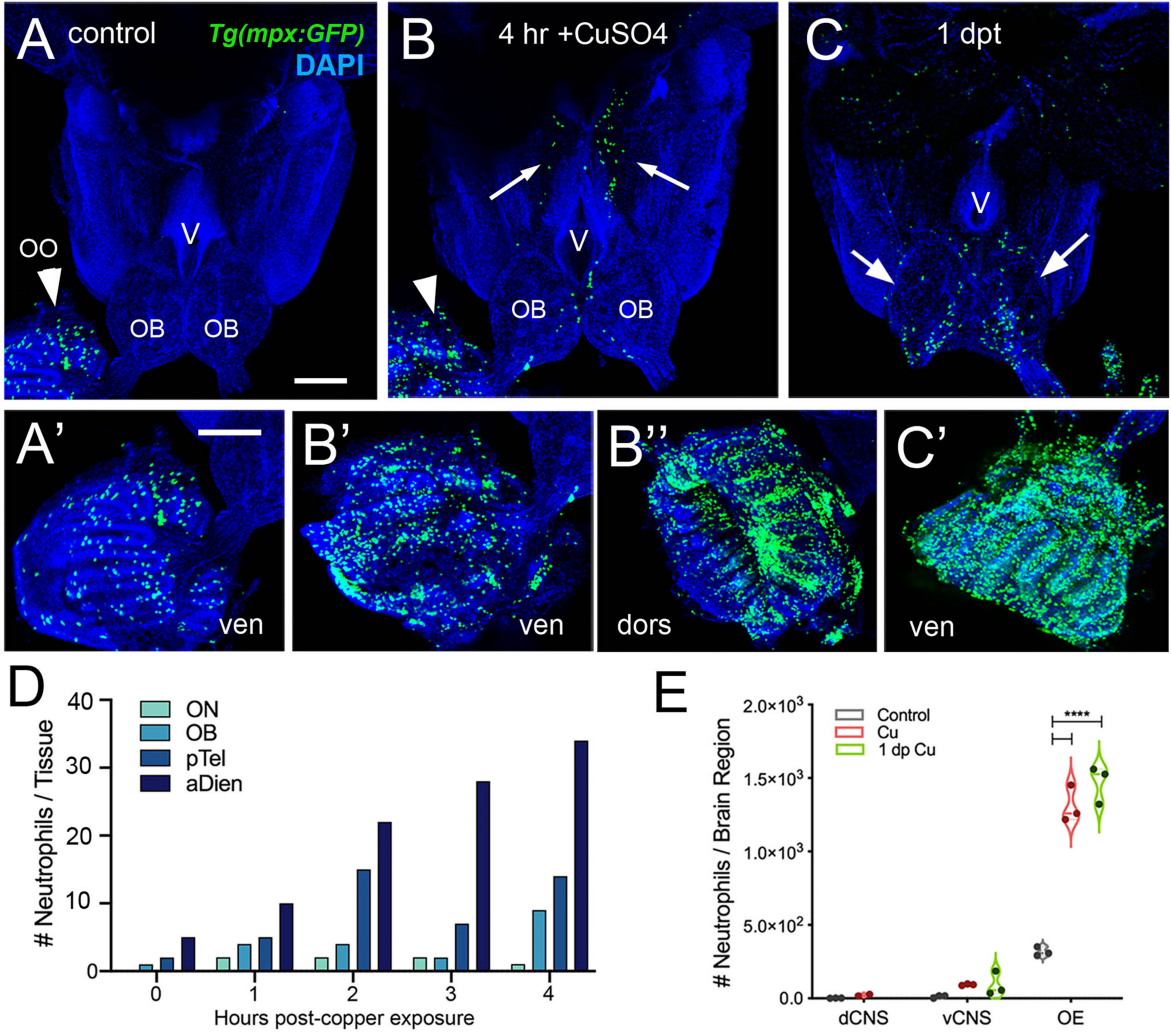
Exposure to copper is correlated with increased neutrophils in the peripheral and central nervous system. **(A–C)** Ventral views of whole mount adult brains from *Tg(mpx:GFP)*. Scale bars: A-C; A’-C’= 100 μm. **(A**, **A’)** Control with neutrophils found only in OO (arrowhead; A’). **(B, B’, B”)** After four-hour exposure to copper, there was an increase in the number of neutrophils in the OO (B, arrowhead, B’, B”). In addition, neutrophils were observed in the ventral OB, along the ventricle (V) and in the ventral telencephalon (**B**, arrows) **(C, C’)** One day post treatment neutrophils were still present in OO **(C’)**, the OB (arrows) and the ventral telencephalon. **(D)** Neutrophils appear over time in an anterior to posterior spatial pattern: olfactory nerve (ON), olfactory bulb (OB), posterior Telencephalon (pTel), and anterior diencephalon; (the numbers for OO are not plotted because number is out of range (average ~1,500, see **E**). **(E)** Copper exposure was correlated with increased neutrophils in OE and ventral CNS. ****: Two-way ANOVA, Tukey’s multiple comparison test, p < 0.05). (****P, 0.0001). **(A–C, A’–C’)** Preparations were selected for imaging based on whether they were intact and the signal to noise of the labeling. **(D)** 1 brain was analyzed per timepoint. **(E)** 3 brains were examined per treatment.

In order to investigate the neutrophil response to damage of the OE, we exposed *Tg(mpx:GFP);Tg(omp:RFP*) adult fish to 10 μM CuSO4. Because of the challenges of live imaging whole mount adult brains, adults were sacrificed at different times after copper exposure to follow the dynamics of neutrophil response over time. In untreated control animals, and consistent with previous results, neutrophils (**Figure 3**, green) were observed only in the OO (**Figure 3A**, arrowhead, A’) and were absent from the brain (**Figure 3A**). After four hours of copper exposure, an increase in neutrophils was observed in the OO (**Figure 3B**, green, arrowhead, B’, B’’). Within the OO the ns and ss OE as well as the EN (**Supplementary Figures 1F, H**) showed an increase in neutrophils in response to damage. Additionally neutrophils were observed in the ventromedial OB, along the telencephalic ventricle (**Figure 3B**, OB, V) and in the ventral telencephalon (**Figure 3B**, green, arrows). Fish left to recover for one day post-treatment still showed elevated numbers of neutrophils in the ventral OB (**Figure 3C**, green, arrows, *D*) and the OO (**Figure 3C’**, green). The increased numbers of neutrophils in the OOs and subsequent appearance of neutrophils in the ventral OB and ventral telencephalon (**Figures 3D, E**, vCNS), suggests that neutrophils may move from the OOs into the ventral CNS in response to peripheral damage.

To further investigate this possibility we used the *Tg(mpx:Dendra2*) line that expresses a phtotoconvertible (green to red) Dendra2 protein in neutrophils (47). The left olfactory organ (OO) of six anesthetized young (2.5 month old) adult fish were exposed to activating light (DAPI filter) (**Supplementary Figures 2, A, A’**) and allowed to recover. Two male and 2 females were exposed to 10 μM CuSO4 for four hours and sacrificed. Tissue was fixed and the CNS examined for the presence of red PC Mpx : Dendra2 positive neutrophils. Four animals, one control (**Supplementary Figures 2 C, C’, C’’**) and three experimental animals (**Supplemental Figures 2D, E**) had red neutrophils in the region of the ventricle. The control animal also showed red photoconverted (PC) Mpx : Dendra2 positive neutrophils in the left posterior OB (**Supplementary Figure 2C’**). The presence of red PC Mpx : Dendra2 positive neutrophils in both control and copper exposed animals suggested that a treatment common to both groups may induce inflammation.

### Damage Induced Changes in Cell Cycle Dynamics in the Olfactory Sensory System

**Figure 4:**
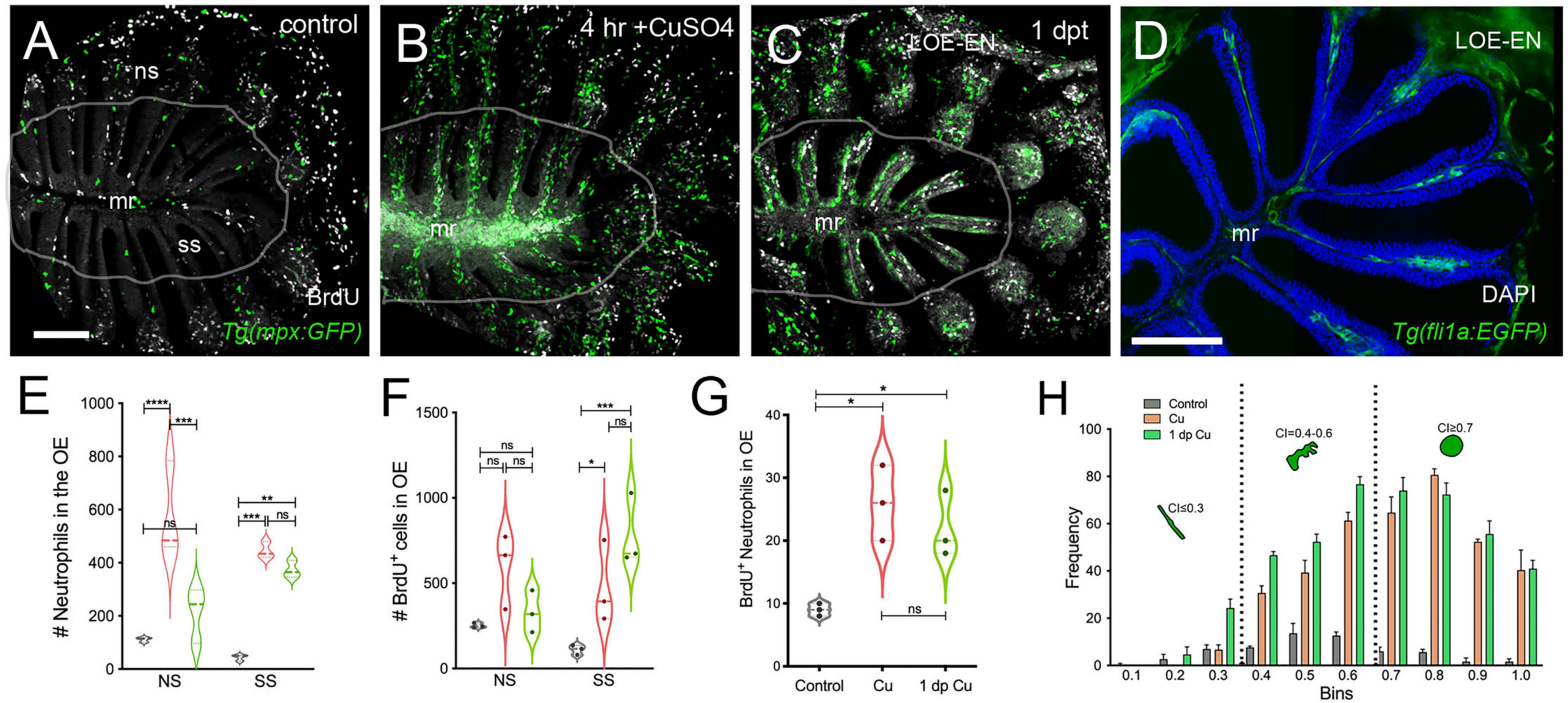
Damage induces cell proliferation of OSN and of neutrophil precursors in the adult olfactory organ. **(A–C)** BrdU labeled cells (white), neutrophils (green) in whole mount OO of adult fish. Scale bars A-C = 50 μm **(A)**. Prior to copper exposure BrdU labeling and scattered neutrophils were observed in the medial raphe (mr), sensory (ss), and non-sensory (ns) epithelia. n= 3 OE. **(B)** After four hours of exposure to copper, intense BrdU labeling was observed in the mr. n= 3 OE. **(C)** One day post recovery, neutrophils lined the lamellae and intense BrdU labeling was observed in ss and LOE-EN. n= 3 OE. **(D)** Section of *Tg(fli1a:EGFP)* adult OE showing extensions of blood vasculature (green) within the OE. Scale bar = 100 μm. n= 3 sectioned heads. **(E)** Significant increases in neutrophil number were observed after 4 hour copper exposure (red) in both the ns and ss epithelia when compared to control (grey). At one dpt (green) only the number of labeled ss cells remained significantly greater than controls. n= 3 OE. **(F)** Damage induced changes in the number of BrdU-positive cells were significant in the ss epithelia but not the ns at 4 hour copper exposure (red) and one dpt (green). n= 3 OE. **(G)** A small but significant increase in mpx:GFP^+^ cells double labeled for BrdU scored in the OE. (E-G, *n*=3 adult OE from different fish; Two-way ANOVA, Tukey multiple comparison test, p < 0.05). (*P, 0.05, **P, 0.01, ***P, 0.001). n= 3 OE. **(H)** Four hours of copper exposure (orange) and one day post-treatment (green), an increase in rounded (nt1; circularity index 0.7 or greater see **Figure 5**) and amoeboid (nt2; circularity index 0.4-0.6) neutrophils were observed. Two-way ANOVA, Tukey’s multiple comparison test, p < 0.05). (*P, 0.05, **P, 0.01, ***P, 0.001, ****P, 0.0001, ns: non significant).

To further investigate the cellular dynamics of the neutrophil response to copper-induced damage in the adult, we repeated the experiments with copper using *Tg(mpx:GFP)* animals in the presence of BrdU. When viewed in flattened whole mount preparations (**Figures 4A–C**), the OE of the adult is organized as a “rosette”, with the central region midline raphe (mr) surrounded by ss and the outer regions of the rosette (tips of the lamellae) containing the ns or respiratory epithelia. In control animals (**Figure 4A**, viewed looking into the rosette) BrdU labeling was observed, consistent with the mitogenic nature of the olfactory system (60, 61). After four hours of copper exposure, BrdU labeling increased significantly in the mr (**Figure 4B**, white, arrow), and in the ns epithelia extending to the EN. In contrast, one day post treatment (dpt) significant increases in BrdU labeling were observed in the ss epithelia (**Figures 4C, F**), consistent with the renewal of OSN in the OE after damage (62). In addition, the neutrophils now lined LOE (**Figure 4C**, green) possibly in association with the BV (**Figure 4D**, green). The number of neutrophils showed significant increases at 4 hours post-treatment (hpt) and remained high in the ss epithelia one dpt (**Figure 4E**; 444.67 ± 31.39 and 373.33 ± 32.32 neutrophils in 4 hpt, red, and 1 dpt, green). Significant increases in BrdU labeling at both 4 hpt and 1 dpt were observed only in the ss epithelia (**Figure 4F**; 480 ± 241.76 and 786 ± 211.6, respectively). Analysis of cells expressing both mpx:GFP and BrdU showed a significant increase compared to control animals (**Figure 4G**, control: 9 ± 1, 4 hpt: 26 ± 6, 1 dpt: 22 ± 5.29). The frequency of rounded (see **Figure 4C**, green, nt1; ci 0.7 or greater) and amoeboid-like (see **Figure 1C**, green, nt2; ci 0.4-0.6) neutrophils, potentially representing “resting” and activated neutrophils respectively, increased in the OE post-damage (**Figure 4H**; **Table 1**). The columnar shaped cells (ci 0.1-0.3) increased in frequency at one dpt in the sensory region (**Figure 4H**, green, 0.2 -0.3 green bars) but remained as the least common morphology. Damage to the OE resulted in an increased number of rounded neutrophils of which a small but significant number were double labeled for BrdU. Thus the majority of the increase in neutrophils was likely due to migration as opposed to proliferation.

**Table 1:** The area under the curve (AUC) and statistical significance of circularity frequency in control, copper, and 1-day post-treatment animals.

Circularity Index	Control	Cu	1 dp Cu
0.1	0.33	0.00	0.00
0.2	2.67	0.00	4.67
0.3	7.00	6.67	24.33
0.4	7.67	30.67	46.67
0.5	13.67	39.33	52.33
0.6	12.67	61.33	76.67
0.7	6.00	64.67	74.00
0.8	5.67	80.67	72.33
0.9	1.67	52.33	55.67
1.0	1.67	40.33	41.00
AUC	5.80 c	35.58 b	41.72 a

Different letters indicate statistical differences between mean values (p<0.05, 1-way ANOVA; a > b > c).

### The Adult Olfactory Sensory System has Extensive Lymphatic Vasculature

**Figure 5:**
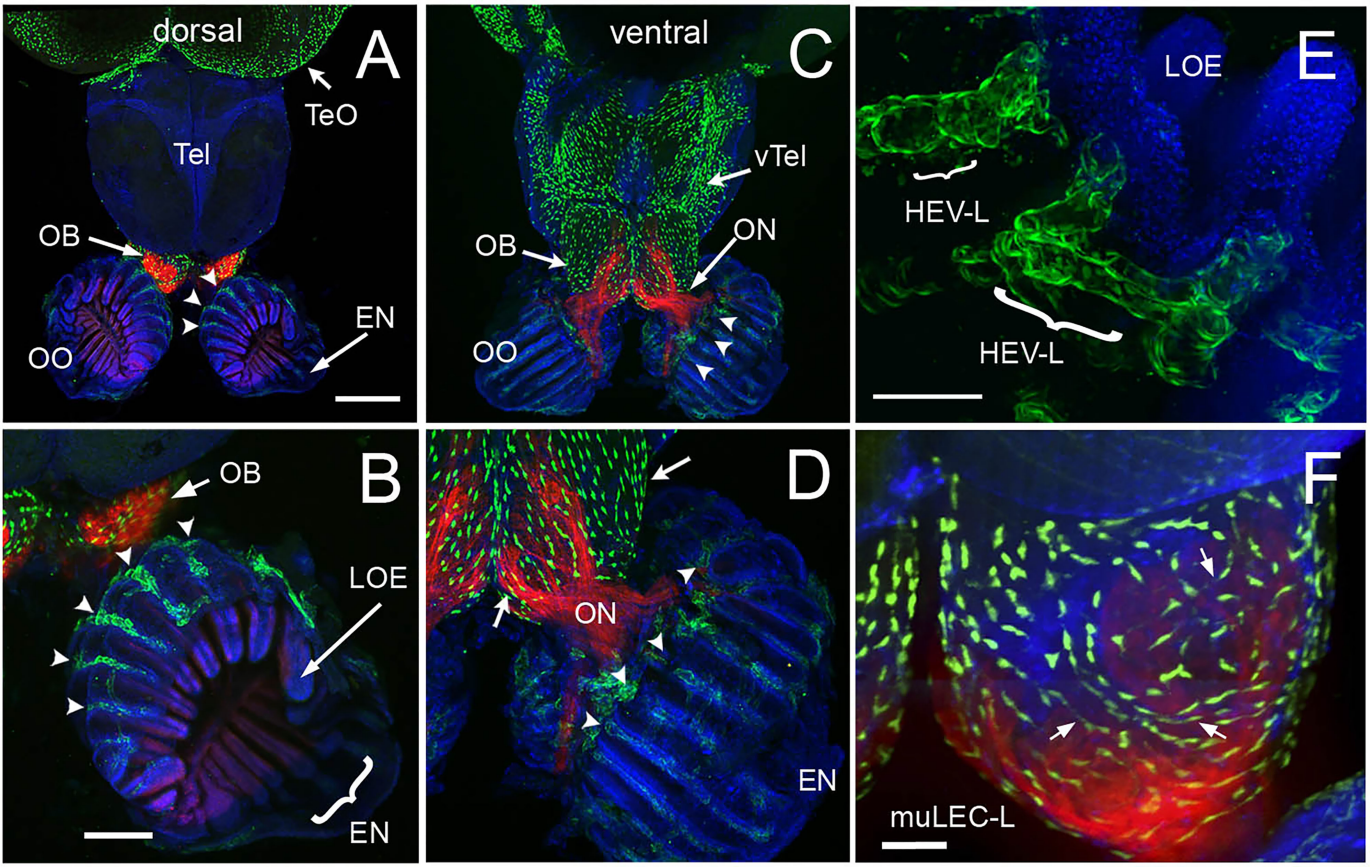
The olfactory organs have an extensive lymphatic vasculature. **(A-F)** Whole mount brains of adult *lyve1b:EGFP;omp:RFP* animals showing OSN (red) and lymphatic vasculature (green). **(A)** The OE and OBs but not dorsal the telencephalon (Tel) showed extensive lymphatic vasculature (LV, green). **(B)** Higher magnification of OO in A with the epineurium (EN, arrow) seen wrapping around the outer surface of lamella of the OE (LOE). Lymphatic cells were found in OO (arrowheads) and OB. **(C)** The LV extended centrally from the OO/OB along the ventral telencephalon (vTel) and posteriorly to the ventral diencephalon. **(D)** Higher magnification of OO in **(B)**. LV (arrowheads) is associated with the olfactory nerve (ON, red) and covers the ventral surface of the OB (green, arrows). **(E)** Lyve1b:EGF^+^ cells in the tips of the LOE resemble High Endothelial Venules (HEVs). **(F)** Putative Mural lymphatic endothelial cells (MuLECs) wrap the OB (arrows). Representative images selected from detailed analysis of 9 brains. DAPI (blue). **(A, C)**= 200 μm; **(B, D)** = 100 μm; **(C, F)** = 50 μm.

**Figure 6:**
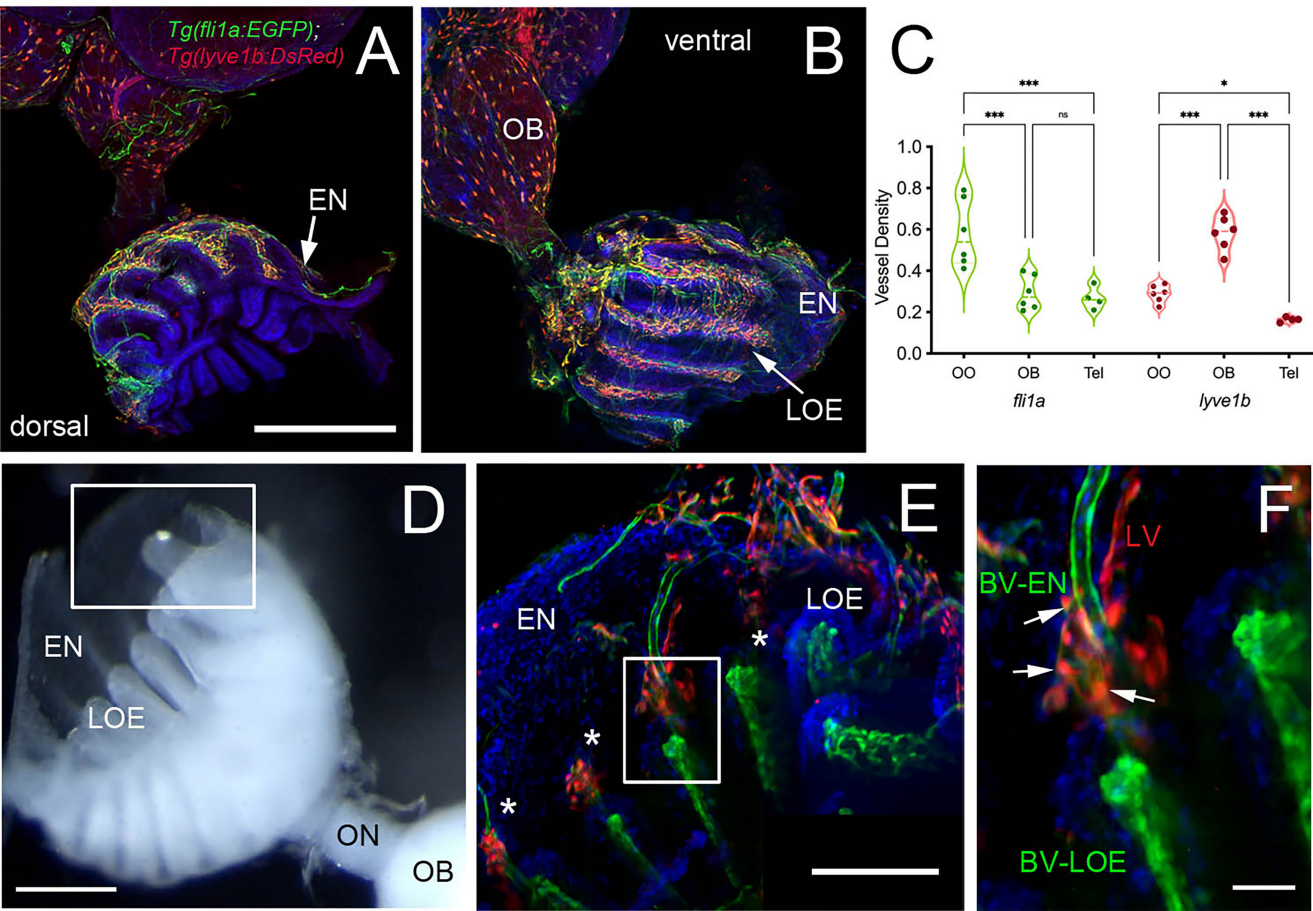
Blood (BV) and Lymphatic (LV) Vasculature wrap the olfactory organs (OO). **(A, B)** Whole mount *Tg(fli1a:EGFP;lyve1b:DsRed)* of the adult OO connected to OB with BV (*fli1a:EGFP*, green) and LV *(lyve1b:DsRed*, red). Dorsal **(A)** and ventral **(B)** views; DAPI (blue), Scale Bars: **(A, B)** = 200 μm. **(C)** BV (red) and LV (green) density is greater in olfactory system (OS = OE and OB) than telencephalon (Tel); (SE, P-value <0.05, unpaired t-test; *n* = 6 adult brains. One-way ANOVA, Tukey multiple comparison test, P < 0.05). Representative images selected from detailed analysis of least 6 brains. **(D)** Transmitted light image of fixed whole mount OO. Boxed area corresponds to area where LOE connects with EN. Scale bar *=* 100 μm, **(E)**. The LV (red) meet the BV (green) at the distal tips of each lamellae (boxed area; asterisks). Scale bar *=* 100 μm, **(F)**. Cells express both lyve1b:DsRed and fli1a:EGFP (arrows). Scale bar = 25 μm. **(A, B)**, **(E, F)**: Analysis of 9 brains. Two-way ANOVA, Tukey’s multiple comparison test, p < 0.05). (*P, 0.05, ***P, 0.001, ns: non significant).

We have previously shown that the lymphatic vasculature (LV) associated with the developing olfactory organs is first evident at 14 days post fertilization (dpf), in the ventrolateral side of the organ (26) To better understand the LV system in the olfactory sensory system of the adult, we dissected brains with olfactory organs attached from *Tg(lyve1b:EGFP;omp:RFP)* animals (**Figure 5**). The olfactory organs (OO) are made up of sensory epithelia containing the omp:RFP^+^ sensory neurons (**Figures 5A–D, F**, red) and respiratory epithelia, surrounded by what appears to be an extension of the epineurium (EN) of the olfactory nerve (**Figures 5A–D**, EN). At this point it is not clear where exactly the meningeal membranes fuse with the epineurium after crossing the cribriform plate (28). Viewed from the dorsal side, lyve1b:EGFP^+^ LV were found in the OO (**Figure 5A**, OO, green, B, green, arrowheads), the olfactory bulb (**Figure 5A**, OB, green, arrow) and the diencephalon (**Figure 1A**, TeO, green, arrow), but not in the telencephalon. In the dorsal OO the lyve1b:EGFP^+^ cells (**Figure 1B**, green, arrowheads) line the lamellae of the OE (**Figure 5B**, LOE). In contrast, when viewed from the ventral side there was an apparently continuous network of LV extending from the OO to the OB and along ventral telencephalon (**Figures 5C, D**, green). The lyve1b:EGFP^+^ cells were also evident in the ventral OO associated with the olfactory nerve (**Figure 5D**, ON, red). Two morphologically distinct lymphatic cell types were observed. Within the OO thick tubular lyve1b:EGFP^+^ cells were associated with the LOE (**Figures 5B, D**, arrowheads), and resemble High Endothelial Venules (HEV-like, HEV-L; **Figure 1E**) that control lymphocyte trafficking in mammals (63); to date these cells have not been described in the peripheral olfactory sensory system. And in the OB, smaller lyve1b:EGFP^+^ cells covering the dorsal OB and ventral telencephalon, apparently connected by fine processes, resembled Mural Lymphatic Endothelial Cells (muLEC-L) after Bower (36) (**Figure 1D**, arrows, green, F, muLEC-L, green). This cell type was also observed in the OO (**Figures 5**, **6**). In contrast to the cells described by Bower, the muLEC-L appeared to be connected by fine processes (**Figures 5, F**, arrows) and not separate cells like the BV-associated muLECs (36). At this time it is not clear whether these connections have a lumen. Thus, in adult zebrafish there is an extensive LV system associated with the olfactory sensory system (**Figure 5**) wrapping the OE (HEV-L), encompassing the olfactory bulb (muLEC-L) with apparently continuous connections along the ventral telencephalon (**Figure 5C**).

To investigate the association of the lymphatic vasculature (LV) with blood vasculature (BV) in the OOs, *Tg(lyve1b:DsRed;fli1a:EGFP)* animals were used to visualize the LV (red) and BV (green) (**Figure 6**). We found extensive BV (fli1a:EGFP^+^) surrounding the OE associated with the EN in both the dorsal (**Figure 6A**, green) and ventral (**Figure 6B**, green) OO and OB. The BV (**Figures 6A, B**, green) and LV (**Figures 6A, B**, red) form an extensive network extending along the lamellae of the dorsal and ventral OE. In comparing the density of BV and LV in the dorsal brain, the OE have a greater density of BV and LV than the OB and telencephalon (**Figure 6C**; **Table 2**, *fli1a*) in contrast to the LV (**Figure 6C**; **Table 2**, *lyve1b*) where the OB has greater density than the OE. The BV (**Figures 6A, B, E, F**, green) and LV (**Figures 6A, B, E, F**, red) extend along the EN that surrounds the LOE (**Figures 6D, E**) and meet at the tips of the LOE where muLEC-L like cells were observed (**Figure 6E** boxed area, red, F arrows). Thus the extensive BV and LV associated with the EN and OE connect along the distal lamellae where distinct BV morphologies are associated with the EN and LOE (**Figure 6F**).

**Table 2:** Mean, standard deviation, and statistical significance of blood and lymphatic vasculature in the olfactory organs (OO), olfactory bulbs (OB), and ventral telencephalon (Tel).

	Tissue	Mean	SD	n
*fli1a:EGFP*	OO	0.581 a	0.163	6
*fli1a:EGFP*	OB	0.293 b	0.083	6
*fli1a:EGFP*	Tel	0.268 b	0.055	4
*lyve1b:DsRed*	OO	0.289 b	0.041	6
*lyve1b:DsRed*	OB	0.584 a	0.082	6
*lyve1b:DsRed*	Tel	0.164 c	0.012	4

Different letters indicate statistical differences between mean values (p<0.05, 1-way ANOVA; a > b > c).

**Figure 7:**
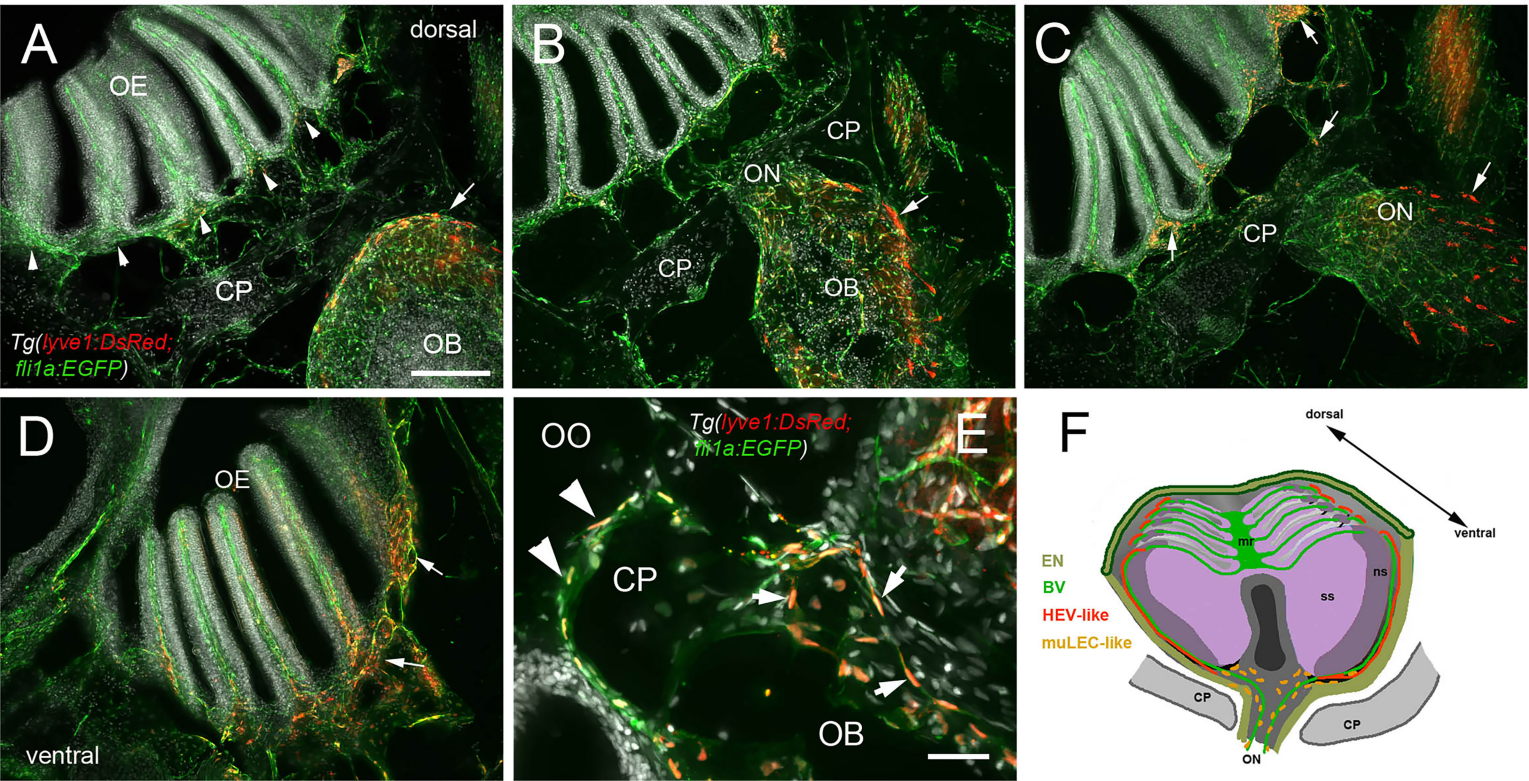
Blood vasculature extends through cribriform plate with muLEC-like lymphatic cells. **(A–E)** Sections from *Tg(lyve1b:DsRed;fli1a:EGFP)* adult brains (n=6 brains examined). (**A)** I. n dorsal sections the OB is separated from the OE by the cribriform plate (CP). The OB has extensive BV (green) extending into the lamellae of the OE and muLEC-like cells (red) on the surface of the OB (arrow). **(A–D)** = 100 μm **(B)**. The ON passes through the CP accompanied by extensive BV (green). muLEC-like cells are on the medial surface (red, arrow) of the OB. **(C)**. The muLEC-like cells (red, arrow) line the ventral side of the ON. **(D)** muLEC-like cells line the basal OE (red, arrows) in the most ventral region of the OO. **(E)** muLEC-like cells on the BV extending across the CP and many are positive for both lyvel1:DsRed and fli1a:EGFP (arrows). DAPI: white. Scale bar = 50 μm. **(F)** Diagram depicting olfactory organ with sensory (ss) and non-sensory (ns) epithelia that have extensive BV (green). The lamellae of the OE contain HEV-like LV (red) that do not extend across the cribriform plate (CP). muLEC-like cells (orange) line the BV and extend from the olfactory bulb across the CP to the basal OE. Scale bars: A-D = 100 μm, E = 50 μm.

In mammals, the olfactory lymphatic route crosses the cribriform plate (CP), which separates the OBs and the OOs, draining cerebral spinal fluid (CSF) through the perineural space surrounding the olfactory nerve (40). This connection to nasal lymphatics carries lymphatic endothelial cells, T, B lymphocytes and antigen presenting cells (APCs) toward the cervical lymph nodes (64). To characterize the LV structure crossing the cribriform plate we sectioned intact, decalcified heads from *Tg(lyve1b:DsRed;fli1a:EGFP)* animals to determine whether the muLEC-L cells or HEV-L cells extended across the cribriform plate (**Figure 7**, CP). Dorsal to, and at the site of, ON crossing (**Figures 7A, B**), the OE was populated primarily by fli1a:EGFP^+^ BV. The lyve1b:DsRed^+^ LV (**Figure 7C**, red, arrowhead) is associated with the fli1a:EGFP^+^ BV surrounding the ON (**Figure 7C**, green) as it crosses the CP and lines the basal region of the OE (**Figures 7C, D**, red, arrows). The muLEC-like cells of the LV lined the BV both on the intra-cranial (**Figure 7E**, arrows) and extra-cranial side (**Figure 7E**, arrowheads) of the ethmoid bone. We never observed HEV-L cells (**Figure 5E**) crossing the CP or on the intra-cranial side of the ethmoid bone. Thus the muLEC-L lymphatic cells associated with the BV were found wrapping the exterior surface of the OB (**Figures 5D, F**), crossing the CP (**Figure 7**) and extending along the EN (**Figures 7A, B**) where they were associated with the HEV-L LV of the olfactory organ (**Figure 7F** *).*

## Discussion

In this study we have shown that the olfactory sensory system has a unique “immune architecture” where neutrophils permanently populate the olfactory sensory organs in association with a complex network of BV-LV. These neutrophils mount a rapid response to copper-induced damage to the OE, populating not only the tissues of the OE and associated EN, but also appearing in tracts extending posteriorly along the ventral CNS. These data demonstrate a role for neutrophils in the olfactory sensory system and suggest that the nasal lymphatic pathway may be a potential site of entry for immune cells into the CNS.

### Neutrophils

It has recently been shown that neutrophils, in addition to their role as the first line of defense in the innate immune response, also transport antigens and populate lymph nodes *via* HEVs where they coordinate early adaptive immune responses (19, 65, 66). Neutrophils are found in many tissues and each such subpopulation performs many functions (67) for instance, the lung is known to retain neutrophils as a host defense niche (68, 69). In mammals the OE is reported to have B lymphocytes, lactoferrin, and lysozyme in the Bowman’s glands (70) and neutrophils in the non-sensory epithelium of the vomeronasal organ (71). In teleosts, limited morphological studies have shown scattered myeloid and lymphoid cells within the OE and *lamina propria* (31, 72, 73). Most recently, in the OO, neutrophils infiltrate and later express neurogenesis-related genes in response to inflammation, suggesting a potential role for neutrophils in the ongoing neurogenesis of the OE (24).

The neutrophils we observed in the OOs were striking not only in their number but also in their limited distribution: they were found only in the OOs and not in the CNS (brain) under normal conditions. After copper exposure there was a large increase in the number of neutrophils in the OE/EN and subsequently neutrophils appeared in the CNS, initially in the ON and ventral lateral OB, and then extending posteriorly along the ventral telencephalon, although far fewer neutrophils were observed in the CNS. This ventral tract from OOs contains a rich network of LV (**Figure 5**), and has previously been suggested as a route for immune cell influx through the basal forebrain in mice (74) and mesenchymal stem cell migration cell from the periphery to the OB (75). Thus, the pattern of neutrophils observed is suggestive of neutrophil migration into the brain from the periphery, along the ON, ventral OB, and ventral telencephalon.

### Neutrophils in CNS

**Figure 8:**
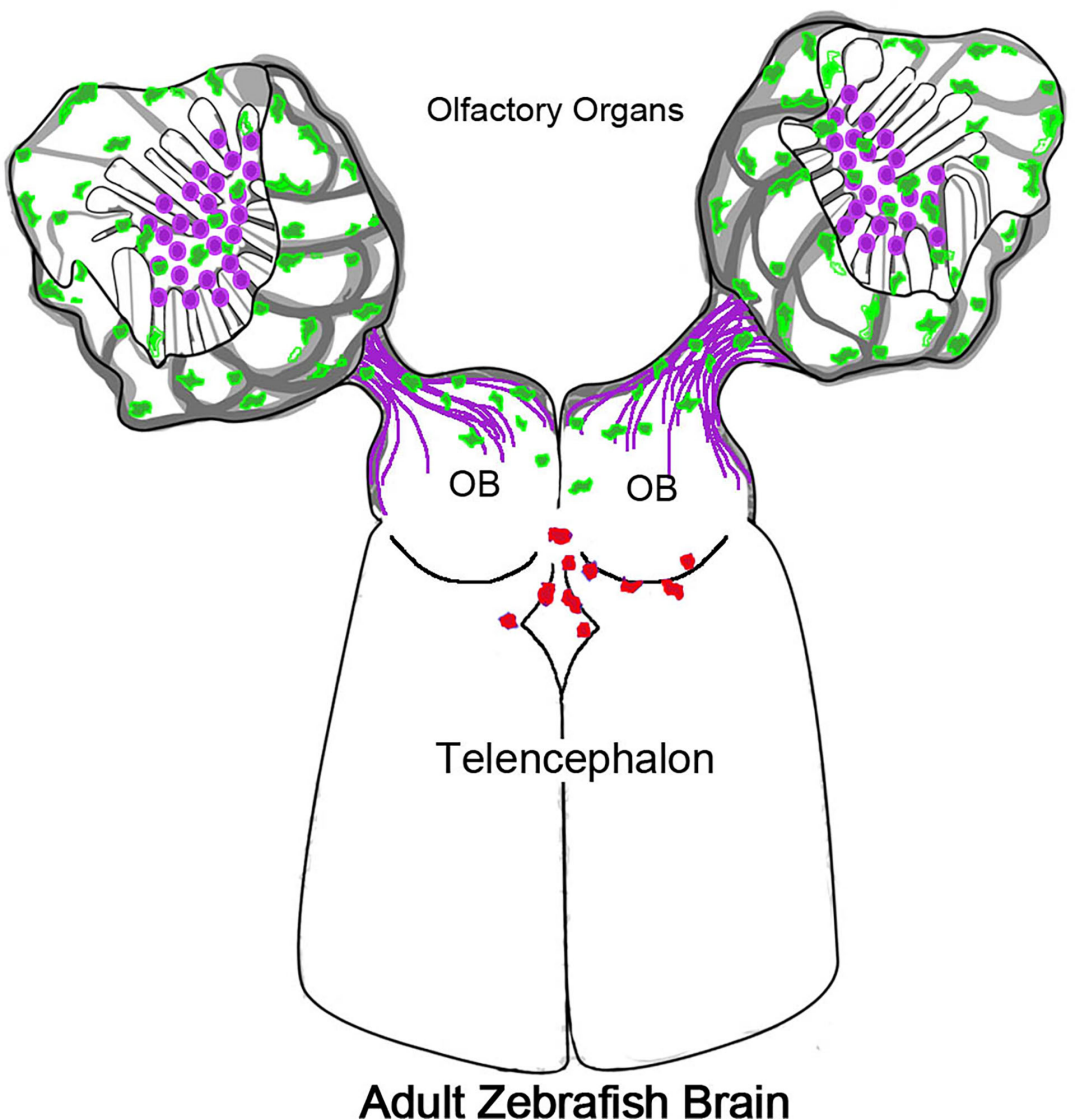
Olfactory organs have unique neutrophil population. Schematic of olfactory organs (OOs), olfactory bulbs (OB) and telencephalon highlighting the association of the neutrophils (green) with the olfactory sensory neurons (purple). Neutrophils PC in the OO appeared in the CNS associated with posterior OB and ventricle.

Experiments using the T*g(mpx:Dendra2*) line where photoconversion was limited to the OO resulted in red (converted) neutrophils in the ventricular region of the telencephalon (**Figure 8**, red cells), support the hypothesis that neutrophils migrate from the OO into the brain. Yet, the presence of red PC-neutrophils in the ventricle region was not specific to copper exposure, as it was observed in both control and experimental animals. Because all animals were anesthetized for the photoconversion process, the most likely explanation is that tricaine (tricaine methanesulfonate/MS-222; the only FDA-approved anesthetic for use in fish in the USA), may affect the OO when the anesthesia is bath applied. Because tricaine is associated with several physiological changes, such as bradycardia, an increased resistance to blood flow through the gill lamellae, and erythrocyte swelling (76), it may have notable effects on the OOs which also contain respiratory tissue. Furthermore, hypoxia, an effect of tricaine anesthesia, has been shown in mammals to promote the infiltration of peripheral immune cells into the brain (77). Thus our results support a model where the olfactory nerve and associated blood-vasculature (26) can serve as a point of entry to the CNS for neutrophils from the OOs. This movement is most likely triggered in response to inflammation but perhaps also in response to odor stimuli. While PC-neutrophils originating in the OO were observed in the region of the ventricle, more experiments are needed to better understand the dynamics of the CNS neutrophils. Deciphering the effects of anesthesia as well as those of odors used in olfactory imprinting (10) on the neural immune response of the OO is a focus of current studies in our lab.

### Lymphatic Vasculature

Evidence supporting a connection between the subarachnoid space of the brain and cervical lymph nodes *via* the nasal mucosa was first proposed over a century ago ]for review see (27, 28)]. In descriptions of the olfactory/nasal drainage in mammals, the LV is generally depicted with terminations at the extra-cranial side of the cribriform plate. Here we found two types of lyve1b:EGFP^+^ LV: one having muLEC like structure where the cells line the BV (36, 37), appeared to be connected, and were found on both the intracranial and extra cranial side of the cribriform plate (**Figure 7**).

A recent study using mouse has confirmed the outflow path of cerebral spinal fluid (CSF) along the olfactory nerves and showed chemical ablation of the OSNs did not increase intracranial pressure suggesting an effect on CSF drainage or production (30). An alternate possibility is that the LV because of its close association with the skull, was unaffected by OSN ablation (see below). Recent studies in zebrafish have described a dorsal meningeal lymphatic network that, like observed in mammals, drains interstitial fluid from the brain and is a route for neutrophil migration, although they move more slowly than when associated with BV (78).

Similar to the tubular lymphatic described in the meninges, we observed a second lyve1b:EGFP^+^ LV with tubular morphology similar to HEVs, in association with the OE/EN on the extra-cranial side of the cribriform plate localized to the distal tips of the LOE. These tubular LV have connections leading away from the region of the respiratory epithelia as has been described for mammals (79) and are severed during our dissections. Preliminary imaging suggests this LV extends across the face of the adult fish and terminates in a region posterior to the eye, and like the dorsal meningeal lymphatic network (78) may also be a candidate region for an (as-yet-undiscovered) analogue of cervical lymph nodes of mammals. Whether these tubular LV observed in the OOs are connected to the skull-associated meninges described in zebrafish is currently unknown.

In mammals the nasal lymphatic route that drains into the cervical lymph nodes through the cribriform plate, carry immune cells such as monocytes, dendritic cells, and T cells (80, 81). Moreover, recently (82) confirmed that cribriform plate lymphatics are a prime site for immune cell drainage and crosstalk upon neuroinflammation (as experimental autoimmune encephalomyelitis), supporting our results on the conserved function of the olfactory lymphatics in regulating immunity. In addition, mammals have Nasal-Associated Lymphoid Tissue (NALT) also referred to as Waldeyer’s lymphatic ring, surrounding the naso/oropharynx. This tissue contains lymphatic vessels and HEVs, which are specialized post-capillary venous swellings, enable lymphocytes circulating in the blood to directly enter a lymph node (by crossing through the HEV).

Recently tissue described as NALT has been reported in fish (83, 84), yet fish do not have lymph nodes. Thus a distinction is made between “organized” NALT and “diffuse NALT” (84) or NALT versus non-NALT (for murine nasal dendritic cells (85) where teleost fish have diffuse-NALT/non-NALT in the olfactory organs. Here we found that the OE/EN has an extensive blood vasculature associated with lyve1b:EGFP^+^ lymphatic endothelial cells resembling high endothelial venules (HEVs) of the lymph nodes in mammals. The HEV-like cells were localized to the tips of the LOE extending on the external side of the EN to the base, terminating in the region where the meningeal membranes fuse on the extra-cranial side of the cribriform plate. The structures observed raise the possibility that in spite of lacking lymph nodes, the zebrafish OOs shows similarities with mammalian lymph node organization, thus suggesting the existence of an organized secondary lymphoid tissue in the OO.

## Conclusions

The surprising finding of neutrophils in the adult olfactory organs resulted from our search to better understand the link between the formation of olfactory memory, as in the case of olfactory imprinting, and its correlation with transcriptional changes in immune associated genes. The extensive network of blood lymphatic vasculature enveloping the OO is associated with neutrophils that appear to traffic within the ventral CNS. Yet neutrophils were also observed within the OOs, including the neural epithelia. The presence of neutrophils in the OOs may be related to the regenerative properties of the OE as the OSNs undergo constant replacement, and/or may represent a special population of secondary lymphoid tissue capable of mounting a rapid immune response. Additionally, perhaps the infiltration of neutrophils can be triggered by specific odors where the communication of horizontal basal cells with infiltrating inflammatory cells (neutrophils) plays a more generalized role in odor recognition and memory.

## Data Availability Statement

The original contributions presented in the study are publicly available. These data can be found here: https://www.ncbi.nlm.nih.gov/geo/query/acc.cgi?acc=GSE196102.

## Ethics Statement

All protocols and procedures employed were reviewed and approved by the Institutional Committee of Bioethics for Research with Experimental Animals, University of Valparaiso (#BA084-2016).

## Author Contributions

KEW designed experiments, analyzed data, wrote the manuscript, and is the guarantor of this work. MFP performed experiments, analyzed data, and reviewed and edited the manuscript. DC performed experiments, CC and GN performed experiments, analyzed data, JT-P performed experiments. All authors contributed to the article and approved the submitted version.

## Funding

National Agency for Research and Development (ANID)- Grants/Fellowships Fondo Nacional de Desarrollo Científico y Tecnológico (FONDECYT) 1160076 (KEW), National Agency for Research and Development (ANID)- Grants/Fellowships Fondo Nacional de Desarrollo Científico yTecnológico (FONDECYT)11190998; ANID- Basal funding for Scientific and Technological Center of Excellence, IMPACT, FB210024 (GN). ANID- Basal funding for Scientific and Technological Center of Excellence, IMPACT, FB210024 (GN); ICM-ANID Instituto Milenio Centro Interdisciplinario de Neurociencias de Valparaíso PO9-022-F, supported by the Millennium Scientific Initiative of the Ministerio de Ciencia (KEW, MFP); CONICYT Doctoral Fellowship (ANID) 21161437 (MFP). The funding bodies did not take part in the design of the study, the collection, analysis, and interpretation of data, or in the writing of the manuscript.

## Conflict of Interest

The authors declare that the research was conducted in the absence of any commercial or financial relationships that could be construed as a potential conflict of interest.

## Publisher’s Note

All claims expressed in this article are solely those of the authors and do not necessarily represent those of their affiliated organizations, or those of the publisher, the editors and the reviewers. Any product that may be evaluated in this article, or claim that may be made by its manufacturer, is not guaranteed or endorsed by the publisher.

## Supplementary Material

Supplementary Figure 1 Copper exposure induces rapid increase in neutrophils in the OOs. **(A)** OSNs (red) in control animal populate the sensory epithelia of the OE. **(B)** Neutrophils in control animal extend up the lamellae and are found in the EN (arrow). **(C)** DAPI labeling in control animal. **(D)** Merge of A-C. 9 brains imaged: representative image from 1 brain. **(E)** Reduced omp:RFP labeling in OO copper exposed animals as neurons die. **(F)** Increase in number of neutrophils in sensory epithelia (ss), non-sensory epithelia (ns) and EN (arrow) of in copper exposed animals. **(G)** DAPI in copper exposed animals. **(H)** Merge of *E-G*. 9 brains imaged: representative image from 1 brain. Scale bar = 100 μm.

Supplementary Figure 2 Photoconverted (PC) neutrophils in *Tg(mpx:Dendra2)* adult fish were observed in the central nervous system. **(A)** Expression of non-photoconverted (non-PC) Mpx : Dendra2 and **A**’ photoconverted (PC) Mpx:dendra2 expressing neutrophils (red) in the left olfactory organ (OO). **(B)** Ventral view of whole mount adult brain from *Tg(mpx:GFP)* from , four-hour exposure to copper. **(C)** Ventral view of whole mount *Tg(mpx:Dendra2)* adult brain showing PC neutrophils (red) in the posterior olfactory bulb (C’, OB) and ventricle (C’’, V). **(D)** PC neutrophils (red, arrow) partially obscured by pigment cell (asterisk). **(E)** Group of PC neutrophils (bracket) in region of ventricle. **(F)** Right OO, Control. Expression of non-PC Mpx : Dendra2 positive neutrophils (green) in wholemount OO. **(F’)** Same preparation viewed in red channel. **(G)** Left OO, PC. Expression Mpx : Dendra2 in wholemount OO showing neutrophils (green) before PC. **(G’)** After PC neutrophils in OO are red. Scale bars: A, A’, F-G’ = 75 μm; B, C= 100 μm; C’-E = 75 μm.

## Abbreviations

(BV) BV, Blood Vasculature; EN, Epineurium: a layer of fibrous connective tissue surrounding the olfactory epithelium; HEV, High Endothelial Venules; LOE, Lamellae of the olfactory epithelia; LV, Lymphatic Vasculature; OO, Olfactory Organ: The complete olfactory structure including the olfactory epithelia and surrounding epineurium, blood vasculature, and lymphatic vasculature; OE, Olfactory Epithelia: The epithelia comprised of sensory (neuronal) and respiratory epithelia, but not epineurium, blood vasculature and lymphatic vasculature.
