# Supplementary figures and images for: The Olfactory Organ Is a Unique Site for Neutrophils in the Brain
Source: Front Immunol. 2022 May 27;13:881702. doi: 10.3389/fimmu.2022.881702 (PMC9186071; doi:10.3389/fimmu.2022.881702)

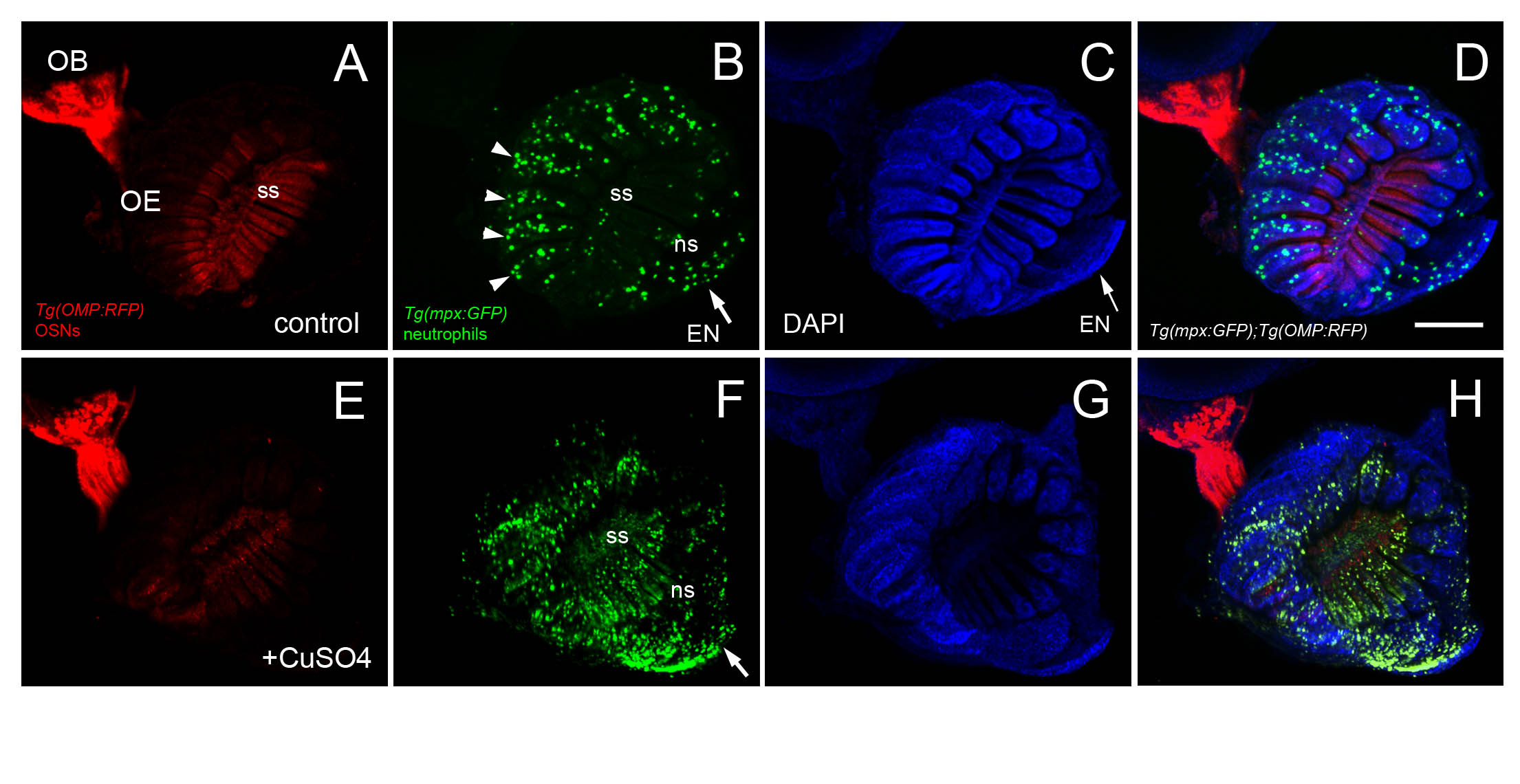

Supplement: Supplementary Figure 1 — Copper exposure induces rapid increase in neutrophils in the OOs. (A) OSNs (red) in control animal populate the sensory epithelia of the OE. (B) Neutrophils in control animal extend up the lamellae and are found in the EN (arrow). (C) DAPI labeling in control animal. (D) Merge of A-C. 9 brains imaged: representative image from 1 brain. (E) Reduced omp:RFP labeling in OO copper exposed animals as neurons die. (F) Increase in number of neutrophils in sensory epithelia (ss), non-sensory epithelia (ns) and EN (arrow) of in copper exposed animals. (G) DAPI in copper exposed animals. (H) Merge of E-G. 9 brains imaged: representative image from 1 brain. Scale bar = 100 μm. [file Image_1.jpeg]

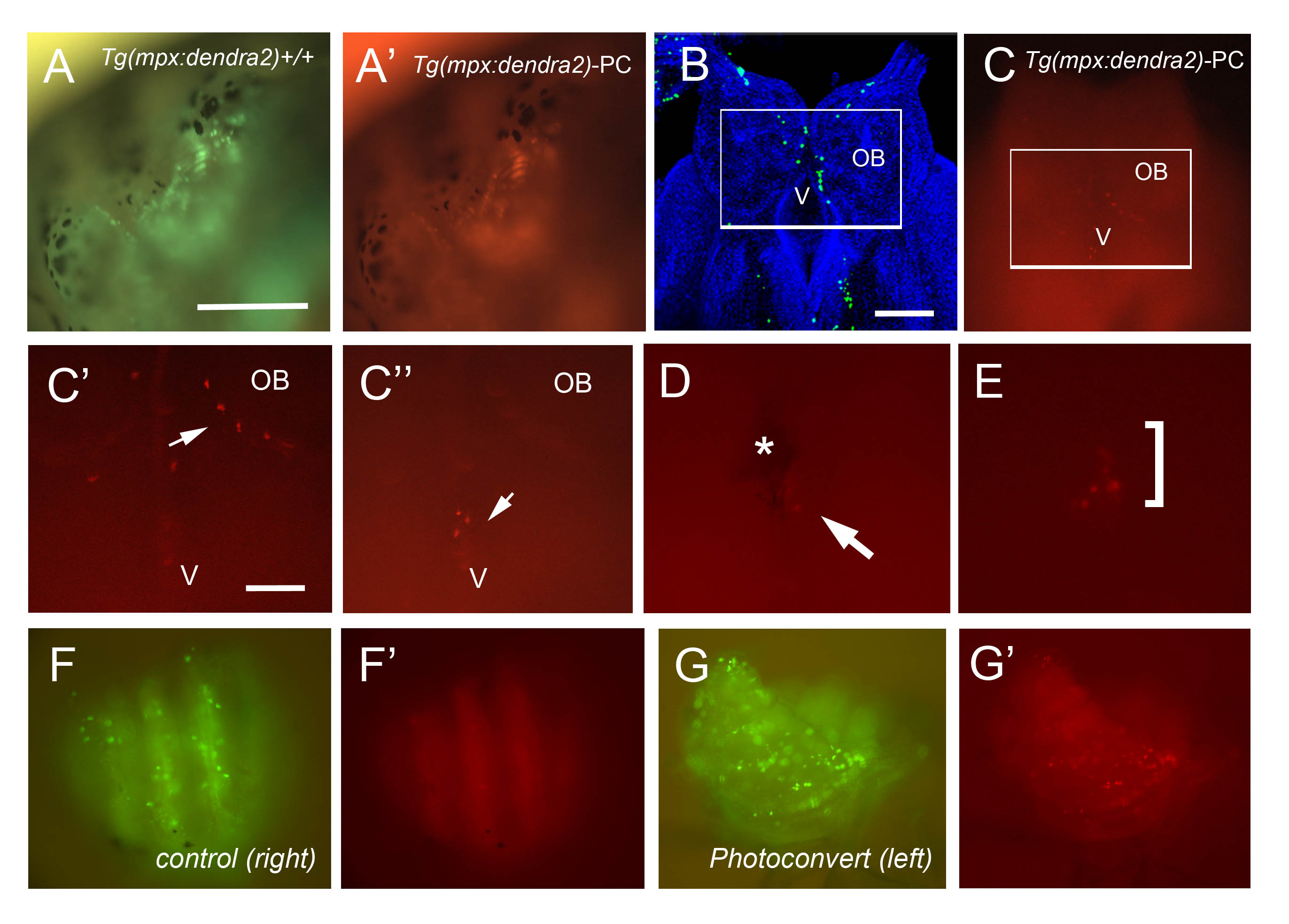

Supplement: Supplementary Figure 2 — Photoconverted (PC) neutrophils in Tg(mpx:Dendra2) adult fish were observed in the central nervous system. (A) Expression of non-photoconverted (non-PC) Mpx : Dendra2 and A’ photoconverted (PC) Mpx:dendra2 expressing neutrophils (red) in the left olfactory organ (OO). (B) Ventral view of whole mount adult brain from Tg(mpx:GFP) from , four-hour exposure to copper. (C) Ventral view of whole mount Tg(mpx:Dendra2) adult brain showing PC neutrophils (red) in the posterior olfactory bulb (C’, OB) and ventricle (C’’, V). (D) PC neutrophils (red, arrow) partially obscured by pigment cell (asterisk). (E) Group of PC neutrophils (bracket) in region of ventricle. (F) Right OO, Control. Expression of non-PC Mpx : Dendra2 positive neutrophils (green) in wholemount OO. (F’) Same preparation viewed in red channel. (G) Left OO, PC. Expression Mpx : Dendra2 in wholemount OO showing neutrophils (green) before PC. (G’) After PC neutrophils in OO are red. Scale bars: A, A’, F-G’ = 75 μm; B, C= 100 μm; C’-E = 75 μm. [file Image_2.jpeg]
